# Phonetic recalibration of speech by text

**DOI:** 10.3758/s13414-015-1034-y

**Published:** 2015-12-24

**Authors:** Mirjam Keetels, Lemmy Schakel, Milene Bonte, Jean Vroomen

**Affiliations:** Department of Cognitive Neuroscience and Neuropsychology, Tilburg University, Tilburg, The Netherlands; Department of Cognitive Neuroscience and Maastricht Brain Imaging Center (M-BIC), Faculty of Psychology and Neuroscience, Maastricht University, Maastricht, The Netherlands

**Keywords:** Phonetic recalibration, Orthographic information, Rapid recalibration, Letters, Speech perception

## Abstract

Listeners adjust their phonetic categories to cope with variations in the speech signal (phonetic recalibration). Previous studies have shown that lipread speech (and word knowledge) can adjust the perception of ambiguous speech and can induce phonetic adjustments (Bertelson, Vroomen, & de Gelder in *Psychological Science, 14*(6), 592–597, 2003; Norris, McQueen, & Cutler in *Cognitive Psychology, 47*(2), 204–238, 2003). We examined whether orthographic information (text) also can induce phonetic recalibration. Experiment 1 showed that after exposure to ambiguous speech sounds halfway between /b/ and /d/ that were combined with text (b or d) participants were more likely to categorize auditory-only test sounds in accordance with the exposed letters. Experiment 2 replicated this effect with a very short exposure phase. These results show that listeners adjust their phonetic boundaries in accordance with disambiguating orthographic information and that these adjustments show a rapid build-up.

## Introduction

We are exposed constantly to unclear speech signals due to, for example, differences in dialects, vague pronunciations, bad articulations, or the presence of background noise. To overcome these ambiguities, speech perception needs to be flexible to adjust to these variations. Therefore, perceivers learn that certain speech sounds should be perceived as belonging to a particular speech-sound category. There is ample evidence that lipread speech (and word knowledge) can be used to adjust our perception of ambiguous speech and can induce phonetic adjustments (Bertelson, Vroomen, & de Gelder, 2003; Norris, McQueen, & Cutler, 2003). This phenomenon has now been replicated many times, but to date it has never been examined whether *orthographic* information (i.e., text), can also serve this role. This is of theoretical importance, because text is culturally acquired, whereas lipread speech has strong biological roots with auditory speech perception (Liberman, 1992).

Substantial research has demonstrated that lipreading affects speech perception. One well-studied example is the McGurk effect in which dubbing /gaga/ lip movements on a /baba/ sound typically results in the fused percept of /dada/ (McGurk & MacDonald, 1976). A McGurk aftereffect also has been reported by Bertelson et al. (2003) who showed that repeated exposure to ambiguous speech sounds dubbed onto videos of a face articulating either /aba/ or /ada/ (henceforth: VbA? or VdA? in which Vb = visual /aba/ and A? = auditory ambiguous) induced perceptual adjustments of this ambiguous sound. Thus, after exposure to VbA? an ambiguous sound was perceived as *more* /b/-like than after exposure to VdA?. The underlying notion is that visual lipread input teaches the auditory system how to interpret sounds, a phenomenon called phonetic recalibration. When auditory nonambiguous and congruent exposure stimuli were used (VbAb and VdAd), no learning effects were observed presumably because there is no intersensory conflict. Rather, aftereffects went in the opposite direction and the ambiguous sound was perceived as *less* /b/-like after exposure to VbAb than VdAd, indicative of “selective speech adaptation” or fatigue (Samuel, 1986; Vroomen, Van Linden, Keetels, De Gelder, & Bertelson, 2004) or a spectral contrast effect (Holt, Lotto, & Kluender, 2000; Holt, Ventura, Rhode, Behesta, & Rinaldo, 2000; Lotto & Kluender, 1998; Lotto, Kluender, & Holt, 1997). Phonetic recalibration by lipread speech has now been replicated many times, also in other laboratories with other tokens and other phonemes. Recent studies show that the phenomenon is phoneme-specific (Reinisch, Wozny, Mitterer, & Holt, 2014) but not speaker-specific (Van der Zande, Jesse, & Cutler, 2014), builds-up fast (Vroomen & Baart, 2009; Vroomen, Van Linden, De Gelder, & Bertelson, 2007), can induce a different interpretation of the same sound simultaneously (Keetels, Pecoraro, & Vroomen, 2015), is comparable for dyslexics (Baart, De Boer-Schellekens, & Vroomen, 2012), and probably involves early cortical auditory networks (Kilian-Hutten, Valente, Vroomen, & Formisano, 2011; Kilian-Hutten, Vroomen, & Formisano, 2011).

Besides lipread-driven phonetic adjustments, *lexical knowledge* can induce phonetic recalibration. Norris et al. (2003) for example demonstrated that exposure to ambiguous sounds embedded in words that normally ended in an /s/ (e.g., *naaldbos*, pine forest) or /f/ (e.g., *witlof*, chicory) resulted in respectively more /s/ or /f/ responses on subsequent ambiguous identification trials (Clarke-Davidson, Luce, & Sawusch, 2008; Eisner & McQueen, 2005; Jesse & McQueen, 2011; Kraljic & Samuel, 2005, 2006, 2007; Myers & Mesite, 2014; Reinisch, Weber, & Mitterer, 2013; Reinisch et al., 2014; Samuel & Kraljic, 2009; Sjerps & McQueen, 2010; Van Linden, Stekelenburg, Tuomainen, & Vroomen, 2007). Van Linden and Vroomen (2007) directly compared lipread and lexically driven recalibration and showed that the effects were comparable in size, build-up, and dissipation rate.

To date, it has never been examined whether these phoneme adjustments also occur when the disambiguating information stems from orthographic information or letters of the alphabet. From an evolutionary point of view, letters are very different from lipread speech or lexical knowledge, because letters are arbitrary cultural artifacts of sound-sight associations that need explicit training during literacy acquisition (Liberman, 1992) whereas for lipread speech there are strong biological constraints between perception and production (Kuhl & Meltzoff, 1982) and the lexicon is acquired in a rather automatic fashion early in life. Therefore, lipread speech and lexical context are both part of the speech signal itself, whereas orthographic information is not, because it only becomes associated with speech during learning to read and occurs together with speech in specific circumstances, such as reading aloud, subtitles, and psychology experiments.

Despite that letters have different biological roots than lipread speech and the lexicon, there is research that demonstrates that co-occurring letters affect perception of speech sounds. In an early study, Frost, Repp, and Katz (1988) showed that words in noise were identified better when matching text was presented rather than nonmatching text (see also Dijkstra, Schreuder, & Frauenfelder, 1989; Massaro, Cohen, & Thompson, 1988). More recently, it has been reported that acoustically degraded words sound “clearer” if a printed word is seen shortly before the word is heard (Sohoglu, Peelle, Carlyon, & Davis, 2014). The specific brain regions responding to letter-speech congruency also have been studied (Froyen, Van Atteveldt, Bonte, & Blomert, 2008; Sohoglu, Peelle, Carlyon, & Davis, 2012; Van Atteveldt, Formisano, Goebel, & Blomert, 2004). For example, Van Atteveldt et al. (2004) reported that heteromodal regions in the STS as well as early and higher-order auditory cortical regions that are typically involved in speech-sound processing showed letter-speech congruency effects. The authors concluded that the integration of letters and speech sounds relies on a neural mechanism that is similar to the mechanism for integrating lipreading with speech (see also Blau, van Atteveldt, Formisano, Goebel, & Blomert, 2008; Froyen et al., 2008; Van Atteveldt, Formisano, Goebel, & Blomert, 2007). Furthermore, letter-sound congruency effects have been shown to be dependent on reading skills as these effects were less evident in dyslexic readers (Blau et al., 2010; Blau, van Atteveldt, Ekkebus, Goebel, & Blomert, 2009; Blomert, 2011; Froyen, Bonte, Van Atteveldt, & Blomert, 2009; Froyen, Willems, & Blomert, 2011; Kronschnabel, Brem, Maurer, & Brandeis, 2014; Zaric et al., 2014). These studies are indicative of a strong functional coupling between processing of letters and speech sounds.

At present, it is unknown whether letter-speech sound combinations induce aftereffects indicative of phonetic recalibration. To study whether letters do indeed induce phonetic recalibration, we adjusted the original exposure-test paradigm (Bertelson et al., 2003). Participants were exposed to ambiguous (A?) or nonambiguous (Ab or Ad) speech sounds combined with printed text (“aba” or “ada”) and then tested with auditory-only sounds near the phoneme boundary. In Experiment 1, participants were exposed to eight audio-visual exposure stimuli followed by six auditory-only test trials (as in Bertelson et al., 2003). In Experiment 2, the exposure phase was reduced to only one stimulus. If letters acted like lipread speech, we expected that exposure to ambiguous speech sounds combined with disambiguating letters would induce phonetic recalibration.

## Experiment 1

### Method

#### Participants

Twenty-two students from Tilburg University participated and received course credits for their participation (18 females; 21.7 years average age). Participants reported normal hearing and normal or corrected-to-normal seeing and were fluent Dutch speakers without a diagnosis of dyslexia. They were tested individually and were unaware of the purpose of the experiment. Written, informed consent was obtained from each participant.

#### Stimuli and materials

**Fig. 1:**
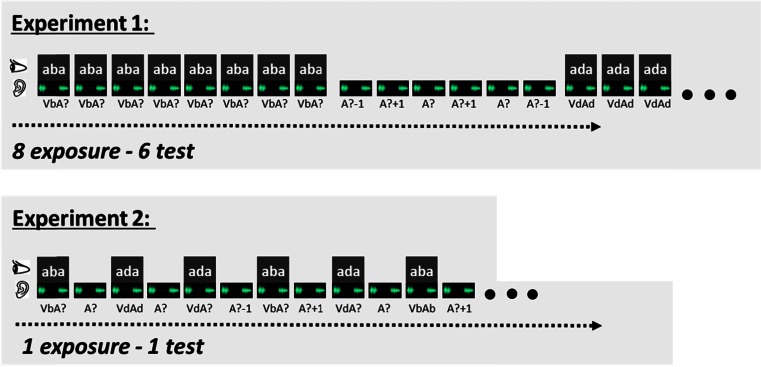
Schematic overview of the Exposure-Test paradigm. In Experiment 1, participants were exposed to 8 auditory-visual exposure stimuli followed by 6 auditory-only test trials (i.e., 8 exposure - 6 test). In Experiment 2, participants were exposed to 1 auditory-visual exposure stimulus followed by 1 auditory-only test trial (i.e., 1 exposure - 1 test).

Participants were seated in front of a 17-inch (600 × 800 pixels) CRT monitor (100-Hz refresh rate) at a distance of approximately 60 cm. The auditory stimuli have been described in detail in Bertelson et al. (2003). In short, we used the audio tracks of a recording of a male Dutch speaker pronouncing the non-words /aba/ and /ada/. The audio was synthesized into a nine-token /aba/–/ada/ continuum by changing the second formant (F2) in eight steps of 39 Mel using the “Praat” speech editor (Boersma & Weenink, 1999). The offset frequency of the first vowel (before the closure) and onset frequency of the second vowel (after the closure) were 1,100 Hz for /aba/ and 1,678 Hz for /ada/ (see Fig. 1 in Vroomen, Van Linden, et al., 2004). The duration of all sound files was 640 ms. From this nine-token continuum, we used the most outer tokens (A1 and A9; henceforth Ab and Ad respectively) and the three middle tokens (A4, A5, and A6; henceforth A?-1, A?, and A?+1, respectively). The audio was delivered binaurally through headphones (Sennheiser HD201) at approximately 66-dB SPL when measured at 5 mm from the earphone.

Visual stimuli consisted of the three letters of the non-words “aba” and “ada” (henceforth Vb and Vd, respectively). The letters were gray (RBG: 128,128,128) presented on a dark background, in the center of the screen (W: 5.5°, H: 2.5°). Visual stimulus duration was 1,200 ms. Letters were presented 450 ms before the sound, because pilot testing showed that this was the most optimal interval to induce perceptual synchrony between the inner speech of the silently read letters (the internal voice that is “heard” while reading) and the externally presented speech sound.

#### Design and procedure

Participants were repeatedly presented with Exposure-Test mini-blocks that each consisted of eight audiovisual exposures followed by six auditory-only test trials. See Fig. 1 for a schematic set-up of the Exposure-Test mini-block design. The exposure stimuli either contained the ambiguous speech sound combined with “aba” or “ada” (VbA? or VdA?) or the nonambiguous speech sound in combination with congruent text (VbAb or VdAd). The interstimulus interval (ISI) between exposure stimuli was 800 ms. The audiovisual exposure phase was followed (after 1,500 ms) by six auditory-only test trials. Test sounds were the most ambiguous token on the continuum (A?), its more “aba-like” neighbor (A?-1), and the more “ada-like” neighbor on the continuum (A?+1). The three test sounds (A?-1; A?; A?+1) were presented twice in random order. The participant’s task was to indicate whether the test sound was /aba/ or /ada/ by pressing a corresponding key on a response box. The intertrial interval (ITI) was 1,250 ms.

Each participant completed 36 Exposure-Test miniblocks where each of the 4 exposure stimuli was presented 9 times (to collect 18 repetitions of each test sound per exposure condition). There was a short pause after 12 mini-blocks. The audiovisual exposure stimuli varied randomly between mini-blocks.

### Results

**Fig. 2:**
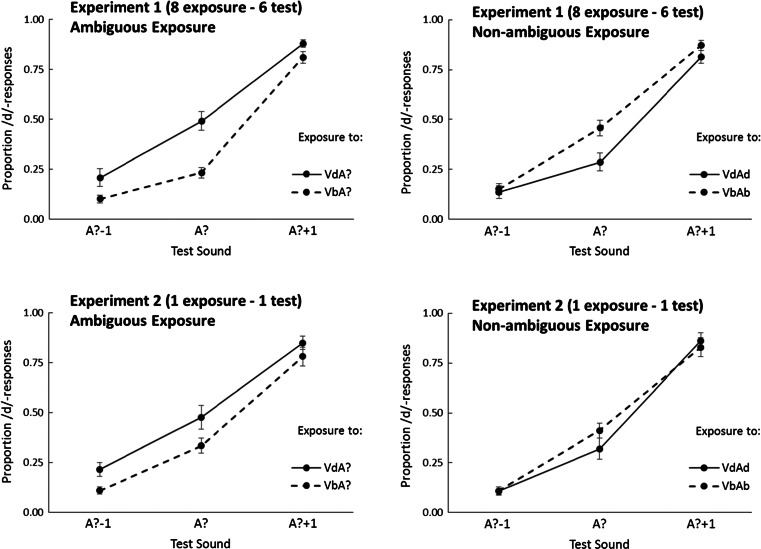
Proportion /d/ responses for the three different test-sounds (A?-1, A?, and A?+1) after ambiguous exposure to VbA? or VdA? (left panel) and nonambiguous exposure to VbAb and VdAd (right panel) for Experiment 1 (upper panels) and Experiment 2 (lower panels). Error bars represent the standard errors of the mean.

The individual proportion of /d/ responses on the auditory-only test trials was calculated for each combination of exposure-sound (ambiguous or nonambiguous), exposure text (Vb or Vd), and test sound (A?-1; A?; A?+1). Figure 2 displays the average proportions of /d/ responses. Most importantly, for ambiguous exposure sounds, there were *more* /d/ responses after exposure to VdA? than after VbA? (Vd-Vb difference = 0.15; indicative of phonetic recalibration). For nonambiguous exposure, there were *fewer* /d/ responses after exposure to VdAd than after VbAb (Vd-Vb difference = −0.08, indicative of selective speech adaptation or spectral contrast effect).

This was confirmed in a generalized linear mixed-effects model with a logistic linking function to account for the dichotomous dependent variable (lme4 package in R version 3.2.2). The dependent variable *Response* was recoded in such a way that a /b/-response was coded as “0” and a /d/-response was coded as “1” (therefore a positive-fitted coefficient reflects more /d/ responses). The factor *Exposure-sound* was recoded into +0.5 for ambiguous, and −0.5 for nonambiguous (therefore the fitted coefficient will correspond to the difference between nonambiguous and ambiguous conditions). Similarly, the factor *Exposure-text* was recoded into +0.5 for Vb, and −0.5 for Vd. The factor *Test-sound* was entered as a numeric factor centered on zero (−1 for A?-1; 0 for A?; and +1 for A?+1) such that the fitted coefficient will correspond to the slope of the /b/-/d/ classification boundary (in units of change in log-odds of /d/-response per one continuum step).

The fitted model included *Response* (/b/ or /d/-response) as the dependent variable, and *Exposure-sound* (ambiguous or nonambiguous), *Exposure-text* (Vb or Vd), *Test-sound* (A?-1; A?; A?+1), and their interactions as fixed factors (see Table 1). The fitted model was: Response ~ 1 + Exposure-sound * Exposure-text * Test-sound + (1 + Exposure-text + Exposure-sound:Exposure-text + Test-sound || Subject)^1^. All the fixed effects correlations were less than 0.2.

**Table 1:** Fitted model: Response ~ 1 + Exposure-sound * Exposure-text * Test-sound + (1 + Exposure-text + Exposure-sound:Exposure-text + Test-sound || Subject)

Experiment	Fixed factor	Estimate	Standard error	z-value	*p*
1	(Intercept)	−0.42	0.14	−2.92	<0.01**
	Exposure-sound	−0.01	0.08	−0.10	0.92
	Exposure-text	−0.17	0.15	−1.07	0.29
	Test-sound	2.28	0.21	11.09	<0.001***
	Exposure-sound*Exposure-text	−1.73	0.23	−7.50	<0.001***
	Exposure-sound*Test-sound	−0.01	0.11	−0.08	0.93
	Exposure-text*Test-sound	0.17	0.11	1.46	0.14
	Exposure-sound*Exposure-text*Test-sound	0.36	0.23	1.58	0.11
2	(Intercept)	−0.45	0.21	−2.12	0.03*
	Exposure-sound	0.21	0.10	2.08	0.04*
	Exposure-text	−0.22	0.25	−0.88	0.38
	Test-Sound	2.57	0.23	11.31	<0.001***
	Exposure-sound*Exposure-text	−1.04	0.26	−4.04	<0.001***
	Exposure-sound*Test-sound	−0.50	0.15	−3.41	<0.001***
	Exposure-text*Test-sound	−0.16	0.16	−1.01	0.31
	Exposure-sound*Exposure-text*Test-sound	0.56	0.30	1.87	0.06

***p < 0.001; **p < 0.01; *p < 0.05.

The analysis revealed a significant negative effect for the intercept (*b* = −0.42, SE = 0.14, *p* < 0.01), which indicates a slight /b/-bias overall. There was no main effect of Exposure-text (*b* = −0.17, *SE* = 0.15, *p* = 0.29) but a significant main effect of Test-sound (*b* = 2.28, *SE* = 0.21, *p* < 0.001) indicative of more /d/ responses for the more /d/-like test sounds. Importantly, a significant interaction between Exposure-sound and Exposure-text was found (*b* = −1.73, *SE* = 0.23, *p* < 0.001), indicating that aftereffects (i.e., Vd-Vb difference) were different for ambiguous and nonambiguous exposure sounds. Significant or nonsignificant effects of any within-subject variables that did not have random slopes are not interpreted.

To further explore the interaction effect between Exposure-sound and Exposure-text, Bonferroni corrected pairwise contrasts were performed and showed a higher proportion of /d/ responses after exposure to VdA? compared with VbA? (data collapsed for the three Test-sounds, *b* = 1.03, *SE* = 0.19, *p* < 0.001). For the nonambiguous Exposure sound, higher proportions of /d/ responses after exposure to VbAb compared with VdAd (*b* = −0.70, *SE* = 0.19, *p* < 0.001). The results of Experiment 1 demonstrate that phonetic recalibration can be induced by orthographic information.

## Experiment 2

In Experiment 2, we further examined whether this effect also can be induced by a very short exposure phase. Evidence for rapid recalibration has been reported before for phonetic recalibration by lipread speech (Vroomen et al., 2007) and for temporal and spatial recalibration (Harvey, Van der Burg, & Alais, 2014; Van der Burg, Alais, & Cass, 2013; Van der Burg & Goodbourn, 2015). Temporal recalibration refers to the phenomenon in which exposure to a certain temporal relation between a sound and a light (i.e., sound-before-light or light-before-sound) results in adjustments of perceived intersensory timing (Fujisaki, Shimojo, Kashino, & Nishida, 2004; Vroomen, Keetels, De Gelder, & Bertelson, 2004). Van der Burg et al. (2013) showed that exposure to only *a single* asynchronous auditory-visual exposure stimulus is sufficient to induce strong temporal recalibration effects afterwards. This led the authors to conclude that temporal recalibration is a fast-acting process that serves to rapidly realign sensory signals (see also Harvey et al., 2014). Similar effects have been reported in the spatial domain by Wozny and Shams (2011), showing that recalibration of perceived auditory space by vision can occur after a single exposure to discrepant auditory-visual stimuli lasting only a few milliseconds. Based on these findings, we hypothesized that phonetic recalibration by orthographic information also might show a rapid build-up. To examine this, participants were exposed to a single audiovisual exposure stimulus followed by an auditory-only test-trial.

### Methods

Twenty-two participants were tested (18 females; 20.1 years average age). Stimuli, procedure, and design were as in Experiment 1, except that an Exposure-Test mini-block consisted of a single exposure-trial (VbA?, VdA?, VbAb, or VdAd) followed by one test-trial (A?-1, A?, or A?+1, randomly varied between mini-blocks). Figure 1 (lower panel) shows the schematic set-up of an Exposure-Test mini-block. Each participant completed 144 Exposure-Test mini-blocks with a short pause after 72 mini-blocks (to collect 12 repetitions per condition).

### Results

After exposure to the ambiguous sounds, there were *more* /d/ responses after exposure to VdA? than after VbA? (Vd-Vb = 0.10, indicative of fast phonetic recalibration), whereas for nonambiguous exposure stimuli there was no difference (Vd-Vb = −0.02).

Data were analyzed as in Experiment 1. The generalized linear mixed-effects model (Response ~ 1 + Exposure-sound * Exposure-text * Test-sound + (1 + Exposure-text + Exposure-sound: Exposure-text + Test-sound || Subject) revealed a significant negative effect for the intercept (*b* = −0.45, SE = 0.21, *p* < 0.05) which indicates a slight /b/-bias overall. There was no main effect of Exposure-text (*b* = −0.22, *SE* = 0.25, *p* = 0.38) but a significant main effect of Test-sound (*b* = 2.57, *SE* = 0.23, *p* < 0.001) indicative of more /d/ responses for the more d-like test sounds. Importantly, a significant interaction between Exposure-sound and Exposure-text was found (*b* = −1.04, *SE* = 0.26, *p* < 0.001), indicating that aftereffects (i.e., Vd-Vb difference) were different for ambiguous and nonambiguous exposure sounds. Significant or nonsignificant effects of any within-subject variables that did not have random slopes are not interpreted.

To further explore the interaction effect between Exposure-sound and Exposure-text, Bonferroni corrected pairwise contrasts were performed and showed a higher proportion of /d/ responses after exposure to VdA? compared with VbA? (data collapsed for the three Test-sounds (*b* = 0.74, *SE* = 0.28, *p* < 0.01). For the nonambiguous Exposure sound, no difference in proportions of /d/ responses were found between exposure to VbAb and VdAd (*b* = −0.30, *SE* = 0.28, *p* = 0.29).

### General discussion

The phoneme boundary between two speech categories is flexible and earlier studies have shown that this boundary can be readjusted by lipread speech or lexical word knowledge that tells what the sound should be (Bertelson et al., 2003; Norris et al., 2003). We report, for the first time, that *orthographic information* (text) also can serve this role. Phonetic recalibration can be induced by letter-speech sound combinations. These adjustments show a fast build-up, even after a single letter-sound exposure. These sound-sight associations that are culturally defined and acquired by extensive reading training can thus adjust auditory perception at the phoneme level.

How does this finding of orthographically induced phonetic recalibration relate to lipread and lexically induced phonetic recalibration? Although the three types of inducer stimuli seem rather different in nature and magnitudes of lipread-induced phonetic recalibration effects seem somewhat bigger (~20-40 % in Bertelson et al., 2003 and Van Linden and Vroomen, 2007) compared with the effects reported in the present study (15 % in Experiment 1 and 10 % in Experiment 2), they appear to rely on a common underlying factor. Van Linden and Vroomen (2007) showed similar characteristics for lipread and lexical-induced recalibration and concluded that the lipread and lexical information serve the same role in phonetic adjustments. The findings of the present study demonstrate that orthographical information also can serve this disambiguating role to induce phonetic adjustments.

We also demonstrated that orthographic phonetic recalibration builds-up very quickly, because it is already stable after a single-exposure stimulus. This finding is in line with previous reports on rapid build-up of phonetic recalibration by lipread stimuli (Vroomen et al., 2007). For temporal and spatial recalibration, a similar fast build-up has been found (Harvey et al., 2014; Van der Burg et al., 2013; Wozny & Shams, 2011). It seems safe to conclude that recalibration behaves similarly such that discrepancies between the senses—either in space, time, or phonetic identity—are rapidly minimized by adjustments of the unreliable source.

Would orthographically induced recalibration be affected when letter-speech sound binding is impaired? Recent studies have reported that impairments in the automatic integration of letters and speech sounds is associated with dyslexia (Blau et al., 2010; Blau et al., 2009; Froyen et al., 2011; Kronschnabel et al., 2014; Zaric et al., 2014; Žaric et al., 2015). Froyen et al. (2011) for example showed in a mismatch-negativity (MMN) paradigm that dyslexics do not exhibit the typical early influences of letters on speech sounds, despite several years of reading instruction. Given that dyslexia has been linked to impairments in grapho-phonological conversions, it might be the case that dyslexic readers do not show phonetic recalibration as normal readers do. This may be an interesting contrast with lipread-induced recalibration, which appears to be in the normal range in people with dyslexia (Baart et al., 2012).

The finding that exposure to nonambiguous speech sounds did not evoke contrast effects in Experiment 2 may appear in conflict with previous findings on selective speech adaptation (Eimas & Corbit, 1973; Samuel, 1986; Vroomen, Keetels, et al., 2004). This null-effect can be explained by the length of the exposure phase, given that selective speech adaptation requires more extensive amounts of exposure.

To summarize, the present study demonstrates that phonetic recalibration can be induced by text very rapidly. Together with previous findings, this is evidence that different information sources (lipread speech, lexical information, text), whether biologically rooted in speech or culturally acquired, can all gain access to the phonetic system.

## Footnotes

1 We used the maximal random effect structure supported by the data (uncorrelated random effects by subjects for intercepts and slopes for Exposure-text, Test-sound, and the Exposure-sound-by-Exposure-text interaction). We determined this random effects structure by starting with a maximal model and removing slopes for fixed effects we are not directly interested in (i.e., random effect correlations, the effect of Exposure-sound and interactions between Test-sound and Exposure-sound/Exposure-text) until the model converged (Bates, Kliegl, Vasishth, in press).
